# Metabolism of Black Carrot Polyphenols during In Vitro Fermentation Is Not Affected by Cellulose or Cell Wall Association

**DOI:** 10.3390/foods9121911

**Published:** 2020-12-21

**Authors:** Gabriele Netzel, Deirdre Mikkelsen, Bernadine M. Flanagan, Michael E. Netzel, Michael J. Gidley, Barbara A. Williams

**Affiliations:** 1 Centre for Nutrition and Food Sciences, Queensland Alliance for Agriculture and Food Innovation, The University of Queensland-St. Lucia Campus, Brisbane, QLD 4072, Australia; g.netzel@uq.edu.au (G.N.); b.flanagan@uq.edu.au (B.M.F.); m.netzel@uq.edu.au (M.E.N.); m.gidley@uq.edu.au (M.J.G.); b.williams@uq.edu.au (B.A.W.); 2 School of Agriculture and Food Sciences, The University of Queensland-St. Lucia Campus, Brisbane, QLD 4072, Australia

**Keywords:** bacterial cellulose, in vitro fermentation, black carrot, polyphenols, anthocyanins, phenolic acids

## Abstract

Fruit and vegetable polyphenols are associated with health benefits, and those not absorbed could be fermented by the gastro-intestinal tract microbiota. Many fermentation studies focus on “pure” polyphenols, rather than those associated with plant cell walls (PCW). Black carrots (BlkC), are an ideal model plant food as their polyphenols bind to PCW with minimal release after gastro-intestinal digestion. BlkC were fractionated into three components—supernatant, pellet after centrifugation, and whole puree. Bacterial cellulose (BCell) was soaked in supernatant (BCell&S) as a model substrate. All substrates were fermented in vitro with a pig faecal inoculum. Gas kinetics, short chain fatty acids, and ammonium production, and changes in anthocyanins and phenolic acids were compared. This study showed that metabolism of BlkC polyphenols during in vitro fermentation was not affected by cellulose/cell wall association. In addition, BCell&S is an appropriate model to represent BlkC fermentation, suggesting the potential to examine fermentability of PCW-associated polyphenols in other fruits/vegetables.

**Highlights**

- Black carrot fractions (puree, supernatant, and pellet) differed in fermentability
- Polyphenol fermentations were comparable between carrots and a cellulose model
- Juice-soaked cellulose can be a model for vegetable polyphenol fermentation

## 1. Introduction

Anthocyanins and phenolic acids are considered to be important components of the health properties of fruits and vegetables [1,2,3,4]. From epidemiological studies and associated meta-analyses in the last two decades, as well as more recent in vivo studies, evidence is accumulating to show that such polyphenol-containing plant foods, when present in the diet, may exert significant health benefits in humans. These include associations with a reduction in the incidence of diabetes, cardiovascular disease, some cancers, and inflammation [2,5,6,7,8,9]. In unprocessed plants, such as fruits or vegetables, polyphenols are present within the plant cell (PC) vacuole, attached to the plant cell wall (PCW), or both [10,11]. This leads to questions concerning their bioaccessibility and subsequent bioavailability, particularly for those compounds which are ingested as part of a plant food, rather than as a purified compound [12].

Bioaccessibility is defined as the amount of an ingested nutrient/dietary compound that is available for absorption in the gastro-intestinal tract after digestion in the small intestine [13,14]. For foods of plant origin, this can be affected by factors such as configuration of the PCW, location within the PCW, and interactions with PCW components [11,12,15,16,17,18,19,20]. Bioavailability, on the other hand, describes the actual amounts of nutrients/dietary compounds which are absorbed from the gastrointestinal tract (GIT) into the bloodstream [18,21]. Polyphenols which are not directly absorbed may subsequently be metabolised by the GIT microbiota [2,22,23], usually in the large intestine. Consequently, there is also growing interest in the properties of their fermentative metabolites, including their potential absorption, interactions with the enterocyte, modulatory effects on the GIT microbiota (stimulating the growth of specific beneficial bacteria thus exhibiting prebiotic properties), and inhibitory effects on inflammatory processes known to be associated with disease states [23,24,25].

Fermentation of a range of different “pure” polyphenols has been investigated, including various anthocyanins and phenolic acids [21,23,26,27,28,29,30,31]. The consequence of in vitro binding of single pure polyphenols to cellulose or isolated cell walls has also recently been reported [32].

Usually, methods for extraction of PCW are harsh, and the resultant product may no longer represent the in planta structure, particularly in relation to delicate interactions between polyphenols and the PCW. The bacterium *Komagataeibacter xylinus* is used as a cellulose biosynthesis model, since it has the same general features of cellulose deposition as found in plants [33]. One advantage of this PCW analogue approach is to avoid destruction of polyphenols (such as by oxidation), as might occur otherwise [11,17,33].

Of the many fruits and vegetable consumed for their high antioxidant properties, black carrots (*Daucus carota* sp. *sativus* var. atrorubens) contain both dietary fibre and polyphenol compounds, such as acylated and non-acylated anthocyanins and phenolic acids [34,35,36,37,38]. Additionally, black carrots are a cheap and substantial source of polyphenols which may be used to enrich food products [39]. It is also known that black carrot anthocyanins and phenolic acids bind to PCW with minimal release after gastric and small intestinal in vitro digestion [40], thus reaching the large intestine intact. To date, digestibility studies have not examined fermentation of non-bioaccessible polyphenols by large intestinal bacteria, which is also required to elucidate the relation between PCW and attached polyphenols in the entire GIT.

In this study, blanched black carrots were fractionated, and the fermentability compared with bacterial cellulose (BCell), both as is, and combined with, carrot juice. The aims were (i) to compare the fermentability of carrot PCW with purified BCell; (ii) to determine whether the carrot PCW versus BCell would make a difference to polyphenolic bioaccessibility; and (iii) to ferment BCell combined with black carrot juice to determine whether such a model of carrot polyphenols attached to pure cellulose, would be an appropriate system to represent the fermentability of black carrots. If so, such a construction could be suitable to examine fermentation of polyphenols and other phytochemicals associated with fruits or vegetables, more generally.

## 2. Materials and Methods

### 2.1. Materials

All solvents were of HPLC grade (Merck Serono Australia Pty Ltd., Kilsyth, VIC, Australia), cy-3-O-glucoside was purchased from ChromaDex (Irvine, CA, USA). Phenolic acid standards were obtained from Sigma-Aldrich (Castle Hill, NSW, Australia). If not otherwise stated, all chemicals and standards were purchased from Merck or Sigma-Aldrich.

### 2.2. Preparation of Substrates

Black carrots (~2 kg) were purchased from a local supermarket (Brisbane, QLD, Australia), washed thoroughly and manually cut into 1 cm pieces. The pieces were then blanched at 90 °C in an equal amount (by weight) of water for 3 min, and drained and cooled to 5 °C. A puree was prepared by blending the pieces in an equal amount of blanching water for 2 min using a domestic blender (BJB840XL, 1800 W, Breville, Botany, NSW, Australia). The puree was then separated into two parts. One part was kept as whole puree (BlkC_WP), the other part was separated into pellet (BlkC_P) and supernatant (BlkC_S) by centrifugation at 18,900× *g* for 15 min at 10 °C (Beckman Coulter, Brea, CA, USA). An aliquot (600 mL) of the supernatant was kept aside for BCell soaking. Following blanching, all procedures were conducted under cool conditions (10 °C).

BlkC_WP, BlkC_P, and the remaining BlkC_S fractions were freeze-dried and cryo-ground (6850 SPEX Freezer/Mill; Metuchen, NJ, USA), using pre-cooling for 5 min, followed by two cycles of grinding at an impactor speed of 10/s for 5 min each, with an intermediate cooling time of 2 min between cycles. All dried black carrot substrates were stored at room temperature in a desiccator pending further use.

Bacterial cellulose was produced as described previously [33]. Briefly, *Komagataeibacter xylinus* strain ATCC 53524 (Cryosite, South Granville, NSW, Australia) was cultivated in HS medium at pH 5.0 [41]. After 72 h incubation at 30 °C, BCell pellicles were harvested and washed in ice-cold sterile Milli-Q water with gentle agitation and frequent rinsing to remove excess medium and bacterial cells. Once purified, the BCell was divided into two portions: (i) pure BCell, and (ii) BCell&S (BCell which was soaked in black carrot supernatant for 12 h, rinsed thoroughly with ice-cold sterile Milli-Q water for 2 h with gentle agitation (100 rpm), to remove unbound polyphenols and sugars). The ratio of BCell to black carrot supernatant (juice) was: 1.96 g (dry weight) of BCell in 500 mL of juice. The pH of the juice was 6.2. All cellulosic materials were freeze-dried, cryo-ground and then stored as described above.

### 2.3. Collection and Preparation of Inoculum (Faecal)

Faecal collection was according to the method of Williams et al. (Williams et al., 2005b). In brief, faeces was collected from five Large White pigs (30–35 kg) fed a semi-purified diet for at least ten days. This diet was based on highly digestible corn starch and fishmeal, and had been formulated to be as free as possible of potentially fermentable carbohydrates, to avoid microbial adaptation to any of the substrates being tested. The pigs were not exposed to antibiotics. All procedures involving the pigs had been approved by the University of Queensland Animal Ethics Committee (NRAVS/244/09/CSIRO).

Following collection, fresh faeces were placed immediately into pre-warmed vacuum flasks pre-flushed with CO_2_, prior to transport to the laboratory. At the laboratory, faeces were mixed with pre-warmed (39 °C), anaerobic, sterile saline (0.9 g L^−1^ NaCl). The ratio between faeces and saline (1:5) was based on the consistency of the faeces, to allow injection into serum bottles. This diluted mixture was homogenised using a hand-mixer for 60 s and strained through four layers of muslin cloth to remove particulates. All procedures were conducted under a constant flow of CO_2_ to maintain a strictly anaerobic environment.

### 2.4. Cumulative Gas Production Technique

Gas production was measured according to the in vitro method described previously [42]. In brief: approximately 0.12 g of each substrate was weighed into replicate 60 mL serum bottles. Two mL of inoculum was added to 38 mL of a semi-purified medium in each bottle, within 2 h of collection. Following inoculation, the bottles were positioned randomly in a pre-warmed incubator at 39 °C. Duplicate bottles for time point intervals at 0, 4, 8, 12, and 24 h were removed, while quadruplicate bottles were fermented for 48 h for end-point analyses. Upon removal, all bottles were immediately placed into an ice-slurry water-bath for 15 min, to inhibit bacterial activity and thereby stop fermentation. Bottles to be analysed for anthocyanins and phenolic acids were then frozen pending sampling. Later, they were thawed and 1mL aliquots collected from all bottles (including the 48 h) and acidified with formic acid (1:1.1) for anthocyanin and phenolic acid analyses. The acidified aliquots were stored at −20 °C until further analysis. One “Blank” (inoculum and medium only) was included, which was not included in the analysis of variance, but the results are shown in the appropriate tables.

### 2.5. Short Chain Fatty Acid (SCFA) and Ammonium Analyses

Short chain fatty acid analysis was conducted according to a previously described method [43]. Samples were thawed, prepared by vacuum distillation, and analysed by gas chromatography (Agilent 6890 Series GC, Agilent Technologies, Wilmington, DE, USA), using a fused silica column (J&W Scientific, supplied by Agilent) with a 1 µm coating. Helium was the carrier gas (flow rate of 6 mL/min). Split injector and FID detector were held at 250 °C. The oven was held at 90 °C for 1 min, then ramped to 190 °C (rate of 10 °C/min) and held for 1 min. SCFA values were corrected to mmoles per gram DM weighed into the serum bottles prior to inoculation.

Ammonium analysis was carried out using a modified colorimetric method [44]. The method is based on the chemical reaction of NH_4_^+^ ions with sodium salicylate and nitroprusside in a weakly alkaline buffer, at a wavelength of 650 nm, using a UV/visible spectrophotometer (Automated Discrete Analyser Model AQ2+, SEAL Analytical Ltd., Fareham, UK).

### 2.6. Sugar Analysis

Samples were quantitatively analysed for sugars using ^1^H NMR in a similar way to a published method [45]. Briefly, 20 mg of dry, ground material was suspended or dissolved at 80 °C overnight in 650 µL D_2_O. When the samples were cooled to room temperature, 100 µL of Sodium 3-(Trimethylsilyl)propionate-2,2,3,3-d4 (TSP) in D_2_O (12 mg/mL) was added as an internal standard and samples were filtered into 5 mm NMR tubes. NMR spectra were measured on a Bruker Avance 500 MHz spectrometer (Bruker, Billerica, MA, USA) operating at 298 K equipped with a 5 mm PABBO probe using a 12 µs 90° pulse, 3.91 s acquisition time, a 20 s relaxation delay and 64 scans. All samples were run a minimum of four times for verification.

### 2.7. Analysis of Anthocyanins and Phenolic Acids

Anthocyanins (cy-3-O-xylglcgal, cy-3-O-xylgal, caffeic acid derivative of cy-3-O-xylglcgal, sinapic acid derivative of cy-3-O-xylglcgal, ferulic acid derivative of cy-3-O-xylglcgal, and p-coumaric acid derivative of cy-3-O-xylglcgal) and phenolic acids (3-O-caffeoylquinic (chlorogenic), 5-O-caffeoylquinic (neochlorogenic), caffeic, sinapic, ferulic and p-coumaric acid) were determined by HPLC-PDA and ESI-LC/MS as described previously [11,17,32].

### 2.8. Data Handling and Statistical Analysis

All gas, SCFA and ammonium data were converted to units per gram DM of weighed-in substrate. Anthocyanins, phenolic acids, and metabolites are expressed as nmol per gram DM weighed-in substrate.

Gas profiles of cumulative gas were fitted to the monophasic model of Groot et al. [46]:

(1) $$\mathrm{DMCV}=\frac{A}{1+{(C/t)}^{B}}$$

where DMCV = cumulative gas produced at time t (mL); A = asymptotic gas production; B = switching characteristic of the curve; C = time (h) at which half of the asymptotic value has been reached—also T½; and t = time (h).

The maximum rate of gas production (R_Max_), and the time at which it occurs (T_RMax_), were calculated using A, B and C values from Equation (1), according to the following equations [47]:

T_Rmax_ = C × (((B − 1)/(B + 1))^(1/B)^) (2)

R_Max_ = (A × (C^B^) B (T_Rmax_^(−B − 1)^))/(1 + (C^B^)T_Rmax_^(−B)^)^2^ (3)

Differences between substrates were tested for significance using Tukey’s studentized range test of multiple comparisons according to

Y = µ + S_i_ + Ɛ_i_ (4)

where Y is the result; μ is the mean; S_i_ is the effect of differences between substrates; and ε_i_ is the error term. The effect of replicate was tested separately, but was not significant (*p* > 0.05) for any of the parameters, and so was removed for the final analysis. Values for blanks (medium and inoculum) were also excluded.

All statistical analyses were performed using the SAS NLIN version 9.1 (curve-fitting) and GLM (significant differences) procedures (Statistical Analysis Systems Institute Inc., Cary, NC, USA).

## 3. Results and Discussion

Percentage dry matter was determined for the substrates prior to fermentation and was as follows: BCell—94.3%; BCell&S—85.5%; BlkC_WP—94.2%; BlkC_S—85.5%; BlkC_P—94.2%; and BlkC_S—87.4%. These values were used to correct all analytical results to /gDM. Table 1 shows a summary of the simple sugar analysis of the substrates.

### 3.1. Concentration of Anthocyanins and Phenolic Acids in Pre-Fermented Substrates

Table 2 shows the concentrations of acylated and non-acylated anthocyanins as well as phenolic acids in the black carrot substrates prior to fermentation.

Total anthocyanins (sum of acylated and non-acylated pigments) were highest in BlkC_S, followed by BCell&S, BlkC_P, and BlkC_WP respectively. A similar distribution pattern was observed for phenolic acids with the highest concentration in BlkC_S, followed by BlkC_WP, BCell&S, and BlkC_P. The acylated and non-acylated anthocyanin profiles found in the experimental black carrot substrates were similar to those reported by others [35,36]. In accordance with a previous report [48], chlorogenic acid (5-caffeoylquinic acid) could be detected as the predominant phenolic acid in all substrates whereas neochlorogenic acid (3-caffeoylquinic acid) was found in much lower concentrations. In addition to the caffeoylquinic acids, four non-esterified hydroxycinnamic acids could be identified and quantified in all substrates: caffeic, ferulic, p-coumaric and sinapic acid. Caffeic and ferulic acid have been reported to occur as free phenolic acids in coloured carrots [48,49], whereas p-coumaric and sinapic acid most likely originated from their corresponding acylated anthocyanins. However, the total amount of these free hydroxycinnamic acids was less than 7.5% of total phenolic acids. It is also important to note that, given the wide variation in DM% values, all anthocyanin and phenolic acid values are reported as per gram DM.

### 3.2. Fermentability of Black Carrot vs. BCell Model System

**Figure 1:**
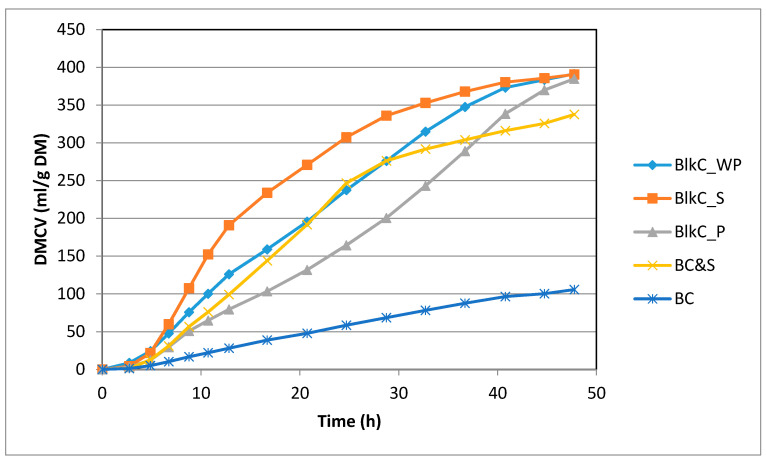
Representative gas profiles for the substrates Black Carrot–whole puree (BlkC_WP), supernatant (BlkC_S), pellet (BlkC_P) and Bacterial Cellulose, alone (BCell) and soaked in black carrot supernatant (BCell&S).

Table 3 and Table 4, and Figure 1, show results for in vitro fermentation parameters, both in terms of kinetics and end-products (48 h).

In terms of fermentation kinetics and end-products (shown in Table 3 and Table 4, and Figure 1), there was no significant difference in terms of fermentation end-products, including total gas and SCFA between the three components of black carrots. There were some differences, however, in the kinetics of fermentation with BlkC_S being the most rapidly fermented, with an earlier ½Time and faster R_max_. Blk-P was slowest and BlkC_WP was intermediate.

This is likely related to the simple sugar data (Table 1), as it can be seen that the supernatant contained about 97% simple sugars, followed by the whole puree and the pellet, comprising 63% and 18% simple sugars respectively. The model BCell&S (soaked in supernatant) was most comparable with the BlkC_P in terms of total sugars, though had less end-product, and faster gas kinetics. BCell, was consistently the least well fermented substrate, having the slowest kinetics, and the least end-products. The presence of almost 17% simple sugars in the BCell&S most likely played a role in the increased fermentation of this substrate compared to BCell. The released non-sugar components from BCell&S (acylated and non-acetylated anthocyanins) may also have been a source of energy for the bacteria.

Overall, the fermentability of the black carrot substrates followed expectations in terms of available energy in the form of carbohydrates. The substrate with the highest sugars, was the most fermentable, while the substrate with the highest cellulose content (BlkC_P) was least fermentable.

### 3.3. Polyphenol Metabolism/Degradation during Fermentation

#### 3.3.1. Anthocyanins

**Figure 2:**
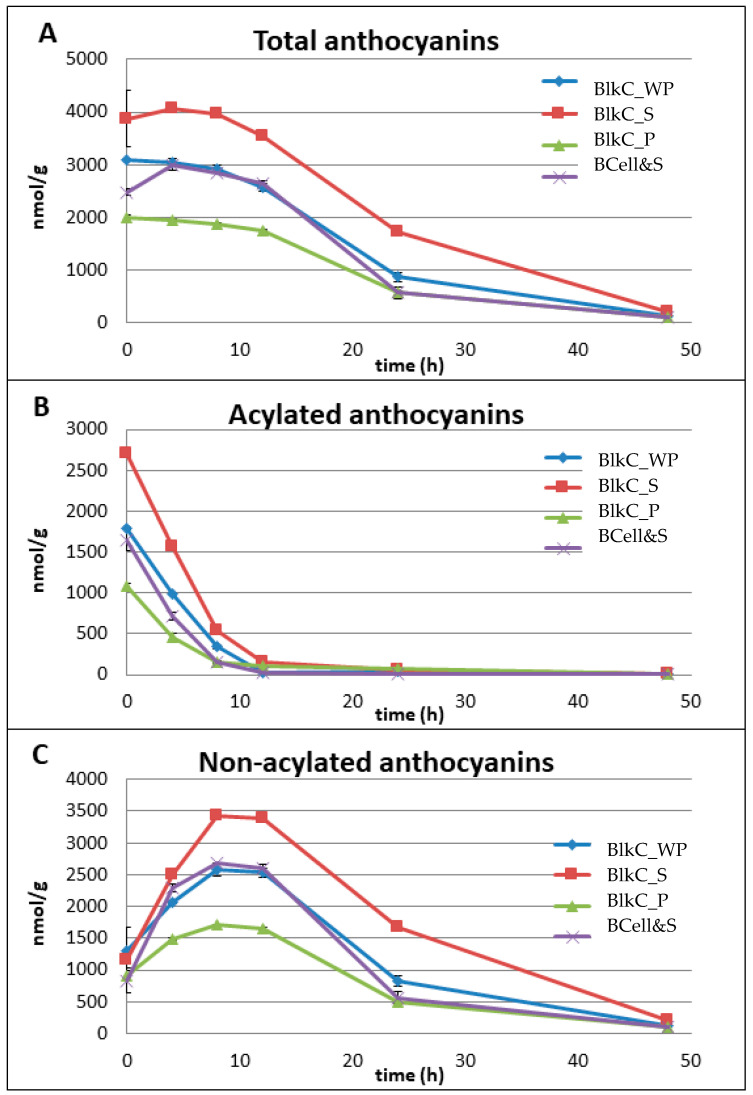
Time course plots for anthocyanins and metabolites for all substrates (time 0 to 48 h); data are means ± SD (*n* = 3); (**A**) total anthocyanins (sum of acylated and non-acylated anthocyanins), (**B**) acylated anthocyanins (sum of caffeic acid derivative of cy-3-O-xylglcgal, sinapic acid derivative of cy-3-O-xylglcgal, ferulic acid derivative of cy-3-O-xylglcgal and p-coumaric acid derivative of cy-3-O-xylglcgal), (**C**) non-acylated anthocyanins (sum of cy-3-O-xylglcgal and cy-3-O-xylgal). The released (free) acylated and non-acylated anthocyanins were analysed by HPLC-PDA and ESI-LC/MS in the post-fermentation supernatants which were collected at each sampling time [11,17,32].

The time-concentration degradation profiles of acylated and non-acylated anthocyanins, as well as total anthocyanins are shown for all substrates in Figure 2. Figure 2A shows the total anthocyanin content of each substrate as a function of fermentation time. An apparent lag phase until 12 h is observed followed by a decline to a very low level after 48 h. However, individual sub-families of anthocyanins may behave differently.

Figure 2B shows the degradation profiles of acylated anthocyanins, indicating a substantial decrease within the first 8 h of fermentation. It seems that the pig inoculum in the present study had comparable metabolic activity to a human faecal suspension used in another in vitro fermentation study [28]. In that study, red radish was used as a source of acylated anthocyanins (p-coumaric, ferulic, caffeic and malonic acid derivatives of pg-3-O-sophoroside-5-O-glucoside). A complete degradation of the acylated red radish anthocyanins via two intermediates (pg-3-O-sophoroside-5-O-glucoside and pg-3-O-sophoroside) to 4-hydroxybenzoic acid as well as the hydroxycinnamates p-coumaric, ferulic and caffeic acid was reported [28], though pg-3-O-sophoroside-5-O-glucoside as the corresponding non-acylated anthocyanin (intermediate) was still detectable at 24 h. A similar degradation pattern was seen in the present study: a substantial decrease of acylated anthocyanins within the first 8 h of fermentation resulted in an almost simultaneous increase of the (corresponding) non-acylated anthocyanins (Figure 2B,C). This increase was likely caused by the generation of cy-3-O-xylglcgal due to the deacylation activity of the microbial inoculum (Supplementary Materials Figure S1). The total amount of acylated cy-3-O-xylglcgal in the black carrot substrates prior to fermentation ranged from 8.5 (BlkC_WP) to 23.1 µmol/g DW (BlkC_S) (Table 4). A “concentration plateau” could be observed between 8 and 12 h, followed by a steady decrease of non-acylated anthocyanins until the end of the fermentation (Figure 2C).

However, it should be noted that low amounts of acylated anthocyanins (<5 nmol/g DM) were still detectable at 48 h of fermentation (Figure 2B). Correa-Betanzo and colleagues also reported low amounts of acetylated anthocyanins after 12 h of fermentation of a crude blueberry extract [50], using human faeces.

The microbial metabolism of non-acylated anthocyanins has been demonstrated previously using a porcine large intestinal inoculum, [51,52], and human faeces [28,45,53]. Despite differences between both in vitro methodologies in terms of the inoculum (i.e., pig or human; inoculum/sample-ratio), incubation time (e.g., 24 or 72 h), and substrates tested (e.g., standard compounds, red wine/grape juice, encapsulated anthocyanins), a similar metabolic pattern was observed in all studies: i.e., an almost complete “disappearance” of all anthocyanins (glycosides) during fermentation most likely from deglycosylation, as well as cleavage of the anthocyanin heterocyclic ring system, resulting in formation of phenolic acids, such as protocatechuic (from cy-3-O-glucoside), syringic (from mv-3-O-glucoside), and vanillic acids (from pn-3-O-glucoside). A significant decrease of non-acylated anthocyanins from 12 h to the end of the fermentation period could be seen in the present study, with up to 200 nmol/g DW (BlkC_S) still detectable at 48 h. Since the analytical procedure was focused on detecting the common black carrot anthocyanins and phenolic acids as well as their degradation profiles, no other phenolic metabolites such as protocatechuic acid were analysed.

In addition to the common acylated and non-acylated black carrot anthocyanins, two other compounds with an intact anthocyanin skeleton could be detected for all substrates during the fermentation process, with concentrations ranging from 128 (BCell&S) to 257 (BlkC_S) nmol/g DW at 48 h. Based on the UV-Vis spectra and mass-spectrometric data (Figure S2) Compound 1 could be tentatively identified as a pg-based glycoside and Compound 2 as a pn-based glycoside. However, neither of these anthocyanin glycosides could be detected at the start of the fermentation. It was demonstrated by Keppler and Humpf that the O-demethylation activity of (pig) caecal microbiota is in contrast to the well-known O-methylation activity of intestinal and hepatic tissues [52]. However, peonidin is a common methylated metabolite of cyanidin. Furthermore, no reports could be found describing the conversion of cy-glycosides to pg-glycosides during in vitro faecal fermentation. It should also be noted that acylated and non-acylated pg- and pn-glycosides were identified as native constituents in some black carrot cultivars [36]. These findings should therefore be interpreted with caution and further investigations are necessary to clarify the origin of these anthocyanin compounds.

#### 3.3.2. Phenolic Acids

**Figure 3:**
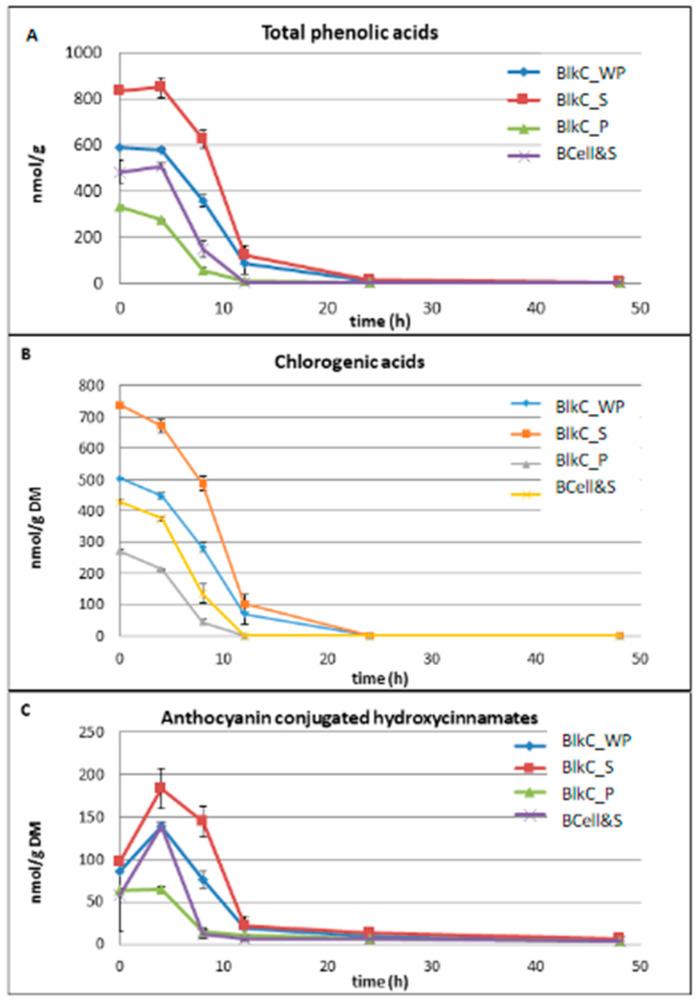
Time course plots for phenolic acids for all substrates (time 0 to 48 h); data shown are means ± SD (*n* = 3); (**A**) total phenolic acids (sum of 5-caffeoylquinic (chlorogenic), 3-caffeoylquinic (neochlorogenic), ferulic, sinapic, caffeic, and p-coumaric acid), (**B**) chlorogenic acids (sum of 5-O-caffeoylquinic, and 3-O-caffeoylquinic acid), and (**C**) anthocyanin conjugated hydroxycinnamates (sum of caffeic, sinapic, ferulic, and p-coumaric acid).

Figure 3 shows the time-concentration degradation profiles of total phenolic acids (A), and the chlorogenic acids (B), as well as the anthocyanin conjugated hydroxycinnamates (C).

The concentrations of free caffeic, ferulic, p-coumaric and sinapic acid, usually bound to cy-3-O-xylglcgal to form the acylated anthocyanin fraction in black carrots, reached a maximum at 4 h of fermentation for all substrates (Figure 3C). This was followed by a substantial degradation within the next 4 h (BlkC_P and BCell&S) and 6 h (BlkC_S and BlkC_WP), respectively. Low concentrations (<5.9 nmol/g DW) of these four hydroxycinnamic acids could still be measured at the end of the fermentation (Figure 3C). The increases in caffeic, ferulic, p-coumaric and sinapic acid within the first 4 h of fermentation were most likely a result of the observed degradation (deacylation) of the acylated anthocyanins to their corresponding non-acylated counterparts (Figure 2C).

The time-concentration degradation profiles of chlorogenic acids were similar for all substrates showing a steady and substantial decrease within the first 12 h of fermentation (Figure 3B). Neither 5-O-caffeoylquinic (chlorogenic) nor 3-O-caffeoylquinic (neochlorogenic) acid was detectable after 24 h of fermentation. These findings are in agreement with earlier reports using a human faecal inoculum [54], which showed an intensive metabolism of caffeic acid and chlorogenic acid with no acids detectable after 2 h. The authors identified 3-hydroxyphenylpropionic acid and benzoic acid as the main microbial metabolites of the fermented hydroxycinnamic acids with a similar metabolic pattern for caffeic acid and chlorogenic acid. However, dihydrocaffeic acid, dihydroferulic acid and another unknown compound were identified as the main metabolites in a batch culture fermentation investigating the impact of coffee and chlorogenic acids on the growth of the human gut microbiota [55]. It has been postulated that there is a “funnelled” metabolic pathway for structurally different phenolic acids (and flavonoids) present in tea and/or red wine/grape juice such as caffeic and p-coumaric acids, in which they are first metabolised to intermediates such as 3,4-dihydroxyphenylpropionic acid and 4-hydroxyphenylpropionic acid and then further to 3-phenylpropionic acid as a metabolic “end product” of the microbial fermentation process [45]. However, the authors also noted that the metabolite profiles varied considerably among the 10 individuals studied, and as a consequence health benefits linked to microbial polyphenol metabolites cannot be generalised.

The time-concentration degradation profiles of bacterial cellulose soaked in black carrot supernatant (BCell&S) and black carrot whole puree (BlkC_WP) were almost identical for acylated and non-acylated anthocyanins, with corresponding total anthocyanins (Figure 2A–C). Similar profiles could be observed for chlorogenic acids (as the main phenolic acids in black carrots), non-esterified hydroxycinnamic acids and total phenolic acids (Figure 3A–C). These findings demonstrate that soaking bacterial cellulose in the “juice” of a fruit or vegetable, may be an appropriate model to examine the fermentability of attached polyphenolic and phenolic compounds.

In 2005, Keppler and Humpf introduced the idea of using the pig as a model system to mimic human microbial polyphenol-metabolism for anthocyanins using commercially available standards [52]. This was followed by work on red wine anthocyanins [51] and flavonol glycosides [56]. Having access to a standardised in vitro model to investigate the microbial metabolism of (controlled) structurally different (poly)phenolics in their natural plant (food) matrix would help to better understand how these compounds are altered by the GIT microbiota. Further in vitro and in vivo studies would be helpful to elucidate the mode of action of black carrots in particular. However, this methodology using an in vitro fermentation technique, and a standard bacterial cellulose combined with supernatants from other fruits and vegetables will be helpful in clarifying how anthocyanins and phenolic acids attached to the PCW, are metabolised by the gut microbiota.

## 4. Conclusions

In vitro fermentations of three fractions of black carrots—the whole puree, the supernatant (juice), and pellet following centrifugation, were investigated. The results showed the effects of the food matrix on disappearance and/or metabolism of anthocyanins (acylated and non-acylated) and phenolic acids, particularly of those polyphenols associated with plant cells (either attached, or trapped within the cell).

## Figures and Tables

**Table 1:** Percentage water extractable sugars *w*/*w* dry matter (mean ± SE) for bacterial cellulose (BCell) and with added black carrot (BlkC) supernatant, and the BlkC fractions (whole puree (WP), supernatant (S), and pellet (P)).

Substrates *	% Sucrose	% Glucose	% Fructose	% Total Sugars **
BCell&S	7.3 ± 0.6	5.9 ± 0.1	3.7 ± 0.2	16.8 ± 0.9
BlkC_WP	39.8 ± 3.1	3.4 ± 0.3	20.5 ± 0.7	63.6 ± 4.1
BlkC_S	59.8 ± 3.2	5.7 ± 0.3	31.9 ± 1.9	97.4 ± 5.4
BlkC_P	10.8 ± 0.6	1.2 ± 0.1	5.9 ± 0.1	17.8 ± 0.8

* BCell alone was not determined, as it is known to have no soluble sugars. ** %Total Sugars is the sum of sucrose, glucose and fructose.

**Table 2:** Anthocyanin and phenolic acid concentrations in the substrates (nmol/g DM) prior to fermentation.

	BlkC_WP	BlkC_S	BlkC_P	BCell&S
Anthocyanins				
Cy-3-O-xylglcgal	319.4 *	1584	655.0	587.3
Cy-3-O-xylgal	1109	5568	2660	2118
Sum (non-acylated)	1428	7152	3315	2705
Caffeic acid derivative of cy-3-O-xylglcgal	96.5	129.0	70.9	66.5
Sinapic acid derivative of cy-3-O-xylglcgal	479.6	948.6	471.8	633.8
Ferulic acid derivative of cy-3-O-xylglcgal	5859	13380	6259.	8059
p-coumaric acid derivative of cy-3-O-xylglcgal	663.0	1496	778.3	860.6
Sum (acylated)	7098	15,954	7580	9620
Total (acylated + non-acylated)	8526	23,106	10,895	12,325
Phenolic acids				
Chlorogenic acids	27009	51052	16075	25597
Non-esterified hydroxycinnamates	1832	3283	1271	1720
Total	28,841	54,336	17,346	27,317
Dry matter (%)	6.7	3.8	12.3	31.5

* Data are arithmetic means, *n* = 3 (CV ≤ 5%); Chlorogenic acids: sum of 5-caffeoylquinic acid and 3-caffeoylquinic acid; Non-esterified hydroxycinnamates: sum of caffeic, sinapic, ferulic and p-coumaric acid.

**Table 3:** Cumulative gas kinetics and end-point parameters for bacterial cellulose alone (BCell) and with added BlkC supernatant (BCell&S), and black carrot (BlkC) fractions (whole puree (WP), supernatant (S), and pellet (P)).

Substrates	n	DMCV (mL)	½ Time (h)	TR_max_ (h)	R_max_ (Lm/h)	pH	NH_4_ *mmol/bottle*
BCell	3	111 ^c^	23.97 ^b^	12.09 ^b^	0.413 ^d^	6.50 ^a^	2.50 ^a^
BCell&S	4	331 ^b^	18.04 ^c^	12.35 ^b^	1.455 ^b^	6.35 ^b^	2.22 ^a^
BlkC_WP	4	377 ^a^	23.10 ^b^	12.96 ^b^	1.445 ^b^	6.37 ^ab^	1.16 ^a^
BlkC_S	4	396 ^a^	15.3 ^c^	9.23 ^b^	1.965 ^a^	6.34 ^b^	2.61 ^a^
BlkC_P	4	394 ^a^	31.9 ^a^	18.43 ^a^	1.205 ^c^	6.37 ^ab^	2.00 ^a^
Probability		<0.0001	<0.0001	<0.0001	<0.0001	0.023	0.58
*MSD*		32.3	2.84	3.76	0.165	0.138	1.34
Blank	2	8.7	20.3	10.29	0.310	6.47	0.30

n: number of replicates analysed, DMCV: cumulative volume per gram DM weighed in (ml), T½: value for “C” from curve-fitting, MSD: minimum significant difference, TR_max_: time of maximum rate of gas production (h), R_max_: maximum rate of gas production (ml/h). Blank: medium + inoculum ^a,b,c,d^ Superscripts differing in the same column, indicate significant differences (*P* < 0.05).

**Table 4:** Fermentation end-products at 48 h for bacterial cellulose (BCell), and black carrots (BlkC) and their fractions (whole puree (WP), supernatant (S), and pellet (P)).

Substrates	n	Acetic	Propionic	Butyric	Total SCFA	%Acet	%Prop	%But	BCR
		mmol/gDM							
BCell	3	2.60 ^b^	0.83 ^d^	0.32 ^c^	4.6 ^c^	56.4 ^b^	18.0 ^e^	7.03 ^a^	0.452 ^a^
BCell&S	3	4.71 ^a^	2.07 ^c^	0.40 ^bc^	7.79 ^b^	60.5 ^a^	26.6 ^c^	5.18 ^c^	0.165 ^b^
BlkC_WP	3	5.79 ^a^	3.33 ^ab^	0.62 ^ab^	10.4 ^ab^	55.5 ^b^	31.9 ^b^	5.94 ^b^	0.137 ^b^
BlkC_S	3	5.63 ^a^	4.22 ^a^	0.67 ^a^	11.3 ^a^	49.9 ^c^	37.4 ^a^	5.90 ^b^	0.139 ^b^
BlkC_P	3	6.17 ^a^	2.42 ^bc^	0.70 ^a^	9.9 ^ab^	62.1 ^a^	24.4 ^d^	7.04 ^a^	0.138 ^b^
Probability		<0.0001	<0.0001	0.0006	0.0002	<0.0001	<0.0001	<0.0001	<0.0001
MSD *		1.49	1.06	0.22	3.01	1.77	1.26	0.61	0.032

n: number of replicates analysed, Acet: acetate, Prop: propionate, But: butyrate, BCR: branched chain ratio, * MSD: minimum significant difference ^a,b,c,d^ Superscripts differing in the same column, indicate significant differences (*P* < 0.05).

## Supplementary Materials

The following are available online at https://www.mdpi.com/2304-8158/9/12/1911/s1. Figure S1: Time course plots for cy-3-O-xylglcgal for all substrates (time 0–48 h). Data are means ± SD (n = 3), Figure S2: Representative UPLC chromatogram of BlkC-WP after 48 h. Detection was performed by UPLC-PDA at 520nm and peak identification was carried out by Orbitrap LCMS [11]. Caffeic acid and p-coumaric acid derivatives of cy-3-O-xylglcgal were not detectable after 48 h.

## Abbreviations

BlkC	Black carrot
BCell	Bacterial cellulose
BCell&S	Bacterial cellulose soaked in supernatant
BlkC_P	Black carrot pellet
BlkC_S	Black carrot supernatant
BlkC_WP	Black carrot whole puree
cy	cyanidin
cy-3-O-xylglcgal	cyanidin-3-O-xylosyl(glucosyl)galactoside
cy-3-O-xylgal	cyanidin-3-O-xylosylgalactoside
DM	Dry matter
GIT	Gastro-intestinal tract
h	Hour(s)
min	Minutes
mv	malvidin
PC	Plant cell
PCW	Plant cell walls
pg	pelargonidin
pn	peonidin
SCFA	Short-chain fatty acids
