# Supplementary material for: Metabolism of Black Carrot Polyphenols during In Vitro Fermentation Is Not Affected by Cellulose or Cell Wall Association
Source: Foods. 2020 Dec 21;9(12):1911. doi: 10.3390/foods9121911 (PMC7766557; doi:10.3390/foods9121911)
Supplement: Supplementary file 1 [file foods-09-01911-s001.pdf]

Supplementary Material

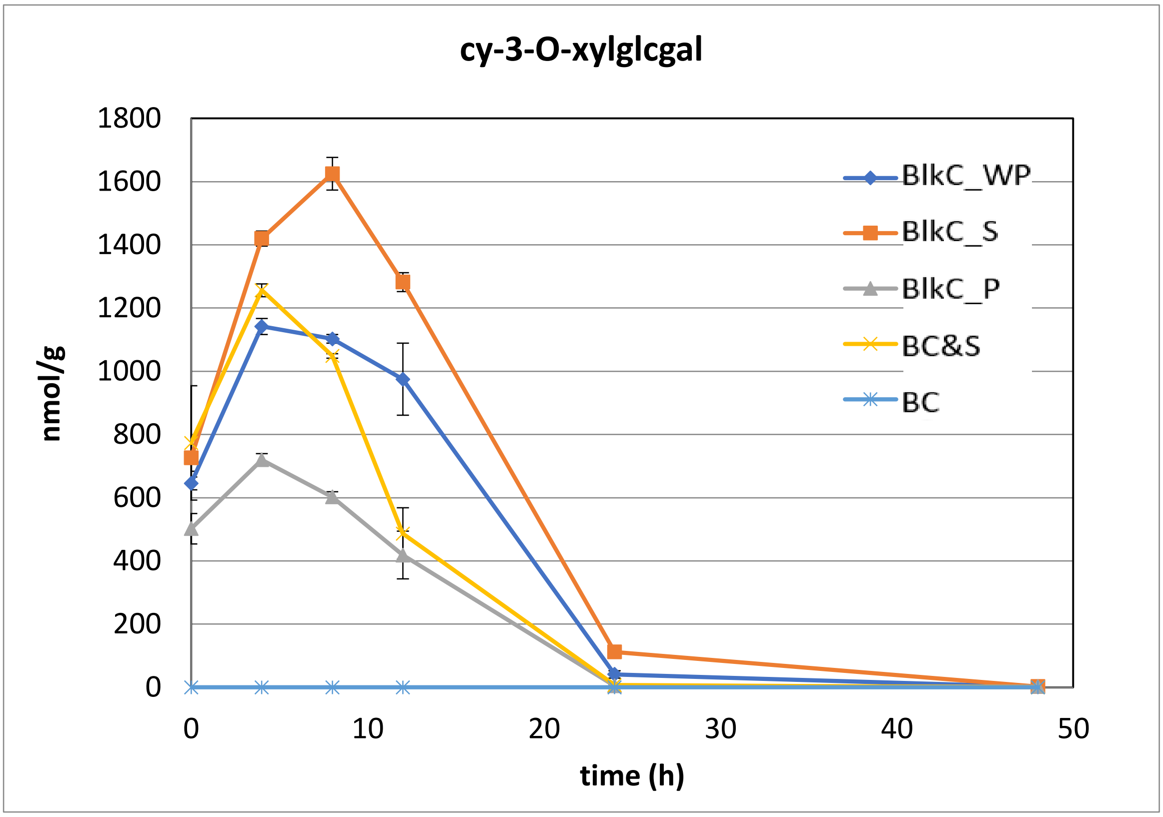

Figure S1. Time course plots for cy-3-O-xylglcgal for all substrates (time 0 – 48h). Data are means ± SD (*n*=3).

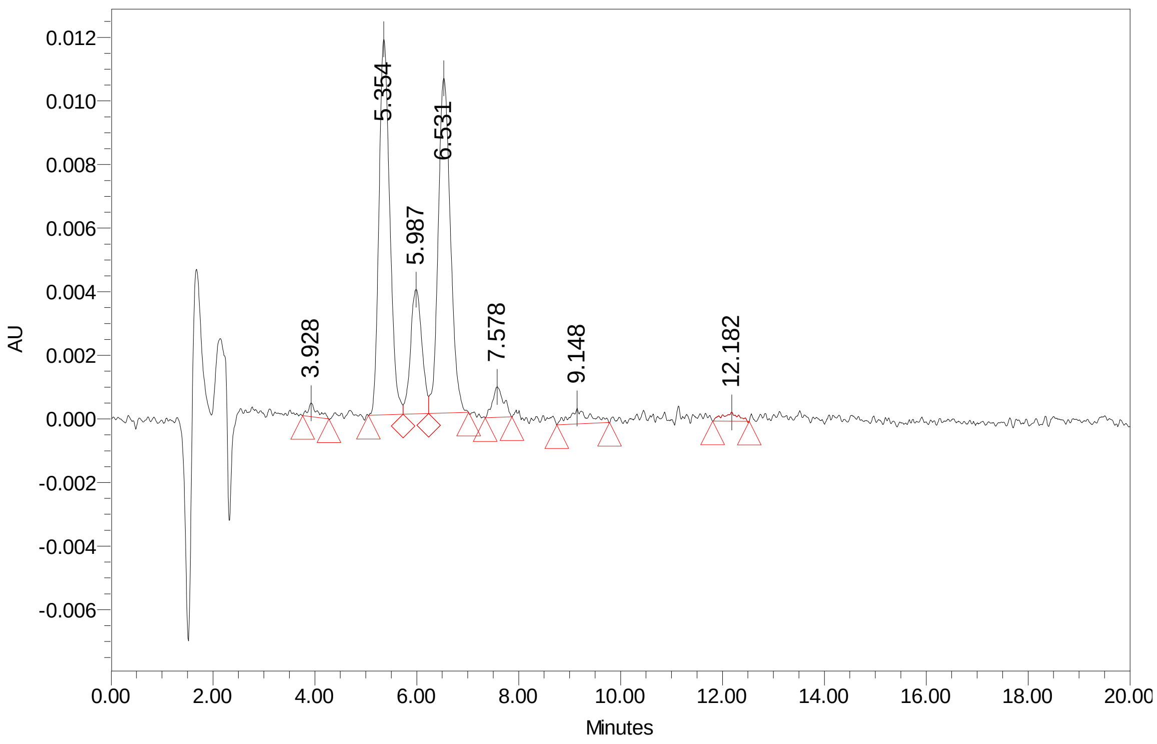

Compounds	Retention Time	*m/z*
cy-3-O-xylglcgal	3.928	743 → 287
cy-3-O-xylgal	5.354	581 → 287
peonidin glycoside based metabolite	5.987	? → 498 → 301
sinapic acid derivative of cy-3-O-xylglcgal	6.532	949 → 287
pelargonidin glycoside based metabolite	7.578	? → 433 →271
ferulic acid derivative of cy-3-O-xylglcgal	9.148	919 → 287

Figure S2. Representative UPLC chromatogram of BlkC-WP after 48 h. Detection was performed by UPLC-PDA at 520nm and peak identification was carried out by Orbitrap LCMS [11]. Caffeic acid and p-coumaric acid derivatives of cy-3-O-xylglcgal were not detectable after 48h.
